# Context-Specific Associations of Physical Activity and Sedentary Behavior With Cognition in Children

**DOI:** 10.1093/aje/kww031

**Published:** 2016-05-24

**Authors:** Daniel Aggio, Lee Smith, Abigail Fisher, Mark Hamer

**Keywords:** cognition, cognitive function, physical activity, sedentary behavior

## Abstract

In the present study, we investigated how overall and specific domains of physical activity and sedentary behavior at the age of 7 years were associated with cognition at the age of 11 years in 8,462 children from the Millennium Cohort Study. Data were collected from 2001 to 2013. Participation in domains of physical activity and sedentary behavior at 7 years of age were reported. Activity levels were also measured objectively. Cognition was assessed using the British Ability Scales. General linear models were used to assess longitudinal associations of physical activity and sedentary behavior, measured both objectively and via self-report, with cognition. Analyses were adjusted for prespecified covariates. Sports/physical activity club attendance (B = 0.6, 95% confidence interval (CI): 0.2, 1.1), doing homework (B = 0.5, 95% CI: 0.0, 0.9), and objectively measured sedentary time (B = 0.8, 95% CI: 0.1, 1.4) at age 7 years were positively associated with cognition at age 11 years in final the models. Television viewing was negatively associated with cognition (B = −1.7, 95% CI: −2.4, −1.0), although the association was attenuated to the null after adjustments for baseline cognition. Objectively measured light physical activity was inversely associated with cognition (B = −0.7, 95% CI: −1.3, −0.1). Moderate-to-vigorous physical activity was also inversely associated with cognition in girls only (B = −1.1, 95% CI: −2.0, −0.3). Associations of physical activity and sedentary behavior with cognition appear to be context-specific in young people.

***Editor's note:*** *An invited commentary on this article appears on page 1083*, *and the authors’ response appears on page 1086.*

The positive association of physical activity with cognitive function and academic achievement in young people has been highlighted in a number of reviews (1–6). School-time physical activity can be substantially increased without detriment to academic performance (1, 2). Executive functioning refers to the complex cognitive processes used for goal-directed behavior (7), broadly including functions such as task-switching, reasoning, planning, and maintaining information in working memory (8), which are particularly relevant for children's learning in various subjects (9, 10). Increasing participation in regular physical activity has been shown primarily to be associated with improved executive function (3). Positive associations have also been reported with working memory (11) and reaction time (12) when using objectively measured physical activity levels. In recent reviews, it has been reported that even acute bouts of physical activity can lead to improved task performance and accuracy and speed of response (3, 13). However, it has been suggested that the benefits of physical activity on cognitive function may be dependent on the type of activity undertaken (14). Physical activities that are challenging and goal-orientated and that involve complex movements and strategic behavior may be more cognitively engaging than repetitive activities, such as jogging, and thus may have a stronger association with cognition (15). Participation in sports is likely to incorporate some, if not all, of these components (i.e., goal-orientated and strategic components) and may therefore be more beneficial to cognitive function than less-structured physical activities.

Equally, associations between sedentary behavior and cognitive function may be context-specific. For example, there is evidence demonstrating the negative association of television viewing with cognitive function and academic achievement (16, 17); however, other domains, such as playing musical instruments and reading and writing, are positively associated with cognitive function (18, 19). Playing video games may also be positively associated with some domains of cognitive function (20).

There have been only a small number of studies in which associations of objectively measured physical activity and sedentary time with cognitive function in young people have been examined (11, 12, 21). Objective assessments provide a more accurate estimate of physical activity volume and intensity, but they do not provide contextual information on the different types of activity. Similarly, they are unable to distinguish between different types of sedentary behavior. Sedentary time may include passive behaviors, such as television viewing, as well as behaviors that require high levels of cognitive engagement, such as doing homework, reading, playing musical instruments, and completing arts and crafts. For these reasons, associations with objective measurement techniques may be challenging to interpret.

In the present study, we aimed to explore how objective and self-reported domains of physical activity and sedentary time are associated with cognitive function using a longitudinal birth cohort study. We hypothesized that context-specific sedentary and active activities at baseline would be more strongly associated with cognitive function at follow-up than a measure of objectively assessed activity (without context).

## METHODS

### Millennium Cohort Study

The Millennium Cohort Study (MCS) is a prospective study of a nationally representative sample of children born at the turn of the century (between September 2000 and January 2002) in the United Kingdom. Eligible children were identified from the records of Child Benefit, a benefit that covers nearly all families in the United Kingdom apart from a small group of children with non-national parents with recent or temporary immigration status (22). Information was collected on 18,818 children at 9 months of age from 1 parent (usually the child's mother). Further surveys were administered at the ages of 3, 5, 7, and 11 years. All measures were collected in the child's home. Web Figure 1 (available at http://aje.oxfordjournals.org/) displays a flowchart of the study measures and when they were taken. Ethical approval was granted by the South West and London Multi-Centre Research Ethics Committees.

### Exposure: physical activity and sedentary behavior

#### Accelerometry-derived activity and sedentary levels

Physical activity and sedentary time were measured objectively using Actigraph GT1M accelerometers (Actigraph, Pensacola, Florida) during the fourth wave of data collection when participants were 7 years of age (between May 2008 and August 2009). Actigraph accelerometers are a valid and reliable way to measure physical activity in young people (23). Full details on the accelerometry procedures have been published previously (24). In brief, accelerometers were delivered by mail to consenting participants and programmed to record data at 15-second intervals (15-second epoch length). Participants were instructed to wear the accelerometers around their waists during waking hours and to take them off during water-based activities for 7 consecutive days. Devices were returned and information was downloaded using Actigraph software. A total of 6,497 children (3,176 boys) met the inclusion criteria, which was set as having at least 2 days with 10 hours or more of wear time (25). Time spent engaging in physical activities of varying intensities were derived using cutpoints generated from a prior calibration study in 7-year-old children (26). Specifically, time sedentary was classified as fewer than 100 counts per minute, and time in moderate-to-vigorous physical activity (MVPA) was classified as more than 2,241 counts per minute (26).

#### Parent-reported behaviors

When children were 7 years old, mothers were asked to report how often per week their children engaged in various domains of physical activity. Questions included how often they participated in clubs or classes involving sports or other physical activities outside of school lessons, such as gymnastics or football. Response options for this question were not at all, less often than once per week, 1 day per week, 2 days per week, 3 days per week, 4 days per week, or 5 or more days per week. Further questions were asked on how often their children were physically active with siblings, how often they were active with a parent, and how frequently they engaged in parent-facilitated visits to the park/playground (not at all, less often than once per month, once or twice per month, once or twice per week, several times per week, or every day or almost every day). These domains of physical activity will hereafter be referred to as “attendance at sports/physical activity clubs,” “sports/physical activity with siblings,” “sports/physical activity with parents,” and “park/playground visits.” Mothers were also asked about their children's participation in a variety of sedentary behaviors, including the number of hours spent watching television/videos/DVDs and using/playing computer or video games (none, less than an hour, 1 hour to less than 3 hours, 3 hours to less than 5 hours, 5 hours to less than 7 hours, or 7 hours or more). Frequency with which the parent draws, paints, or makes things with child (not at all, less often than once per month, once or twice per month, once or twice per week, several times per week, or every day or almost every day) and frequency with which the child reads for enjoyment on their own excluding schoolwork (every day or almost every day, several times per week, once or twice per week, at least once per month, every few months, at least once per year, or less often [than once per year] or never) were reported. Finally, mothers reported how many minutes their child typically spent per week doing homework. These domains of sedentary behavior will hereafter be referred to as “television viewing,” “playing computer/electronic games,” “reading,” “doing arts and crafts,” and “doing homework.” Although these specific questions have not been validated, previous studies have shown that similar parent-reported physical activity and sedentary behavior measures are valid (27) and reliable (28).

### Cognitive function

Cognitive functioning has been assessed in the Millennium Cohort Study since the second wave of data collection (3 years of age) through a battery of assessments, including a number of tests from the British Ability Scales (BAS) (29). When the children were 3 years old, the tests included the BAS Naming Vocabulary scale, which is used to assess the spoken vocabulary of children. This was accompanied at age 5 years with the BAS Picture Similarities and Pattern Construction scales, which are used to assess problem-solving skills and spatial awareness, respectively. When the children were 7 years old (sweep 4), the assessments again included the Pattern Construction scale, as well as BAS Word Reading scale. The Word Reading scale is used to assess verbal ability. When the children were 11 years old (sweep 5), a different scale was used again in the form of the BAS Verbal Similarities test, which is used to examine verbal reasoning and verbal knowledge. *T* scores or standardized BAS scores were generated using BAS normative data set for each test (30), with a higher score indicating better performance. The raw scores are converted into *T* scores except for those from the Word Reading scale. A participant with a *T* score of 50 had the same raw score as the mean of those in their norming group. A *T* score of 60 represents a score that is 1 standard deviation above the mean of their norming group. Standardized scores for the Word Reading scale were transformed in the same manner, but they do not have a mean of 50 and standard deviation of 10. The BAS measures are nationally standardized and have shown good inter-rater reliability (31).

### Covariates

Our choice of covariates was theory-driven based on existing evidence in the field (32). We adjusted for baseline cognitive function prior to age 11 years. Because the majority of BAS scores at ages 3–7 years correlated strongly with each other (see Web Table 1), we constructed a composite baseline BAS score by calculating the mean of all standardized test scores at ages 3, 5, and 7 years. In addition, a number of confounding factors potentially associated with both the exposure and the outcome were identified before the analyses, including child's sex, ethnicity (white, mixed, Indian, Pakistani/Bangladeshi, black, or other), Actigraph wear time (for accelerometry analyses only), maternal highest academic qualification (National Vocational Qualification level 1–5), maternal age, income (above or in poverty), maternal smoking status during pregnancy (smoker or nonsmoker) and Strengths and Difficulties Questionnaire score (33). Parents completed the Strengths and Difficulties Questionnaire when their child was 7 years old. It is considered a valid and reliable measure of child behavioral and emotional problems (33).

### Statistical analysis

Participants were categorized into 3 equal groups for each domain of parent-reported physical activity and sedentary behavior (low, medium, and high). Likewise, participants were also categorized for time in each objectively measured activity level. General linear models were then used to determine how specific domains and time in specific intensities of activity at age 7 years were associated with BAS scores at age 11 years. To understand the importance of the context of the activity, we repeated the accelerometry analysis stratifying first for time spent reading (low vs. high) and then for attendance at sports/physical activity clubs or classes (low vs. high). Further, boys and girls tend to differ with regard to the types of activity in which they typically engage (34), which may consequently affect associations with cognitive function; we therefore also stratified by sex. Model 1 was adjusted for sex and also time spent wearing the device (minutes per day) in analyses in which we used accelerometry variables as the main exposure. Model 2 additionally included the composite baseline BAS score with adjustment for cognitive function at ages 3, 5, and 7 years. Model 3 was further adjusted for ethnicity, maternal age, maternal qualifications, income, smoking status during pregnancy, and Strengths and Difficulties Questionnaire score. All analyses were conducted in SPSS, version 22 (IBM Corp., Armonk, New York).

## RESULTS

The final sample consisted of 8,462 children with complete parent-reported data at age 7 years accompanied by complete cognitive data at ages 3, 5, 7, and 11 years. A total of 4,724 participants also had valid accelerometry data for analyses. Those in the accelerometry sample (*n* = 4,724) differed significantly from those who were excluded from that analysis (*n* = 3,738) in terms of BAS Verbal Similarities scale scores, ethnicity, Strengths and Difficulties Questionnaire scores, and parental factors. Differences between these samples are presented in Web Table 2. Sample characteristics are presented in Table 1, including accelerometry and BAS Verbal Similarities scale scores. Approximately 80% of participants wore the accelerometers for 5 days or more, and a large proportion also had at least 1 valid weekend day (79%). On average, participants spent 62.1 minutes/day in MVPA, with 48.8% meeting current physical activity guidelines (≥60 minutes/day of MVPA).

**Table 1.** Descriptive Statistics of Participants at Age 7 Years and Score on the British Ability Scales Verbal Similarities Scale at Age 11 Years, Millennium Cohort Study, 2001–2013

Characteristic	No.	%	Mean (SD)
Total sample	8,462		
Male sex	4,228	50.0	
White British race/ethnicity	7,468	88.3	
Maternal age, years			36.6 (5.8)
Maternal academic qualification level ≥4^a^	3,547	41.9	
Poverty-level income	2,013	23.8	
Smoked during pregnancy	2,649	31.3	
Strengths and Difficulties Questionnaire score			7.3 (5.0)
BAS Verbal Similarities scale score			59.5 (9.5)
Accelerometry sample	4,724		
Sedentary time, minutes/day			392.7 (65.9)
Light activity, minutes/day			279.9 (40.6)
MVPA, minutes/day			62.1 (22.1)
Accelerometer wear time, minutes/day			734.6 (59.7)

Abbreviations: BAS, British Ability Scale; MVPA, moderate-to-vigorous physical activity; SD, standard deviation.

^a^ National Vocational Qualification score.

### Longitudinal associations

The final models (model 3) showed that attendance at sports/physical activity clubs and doing homework at age 7 years were positively associated with BAS Verbal Similarities scale score at age 11 years (see Tables 2 and 3). The final models also showed an inverse association between sports/physical activity with siblings and BAS Verbal Similarities scale score. The initial models showed that television viewing was inversely associated with Verbal Similarities scale scores, but this association did not persist after controlling for previous cognition levels. The initial and intermediate models showed a positive association between reading and Verbal Similarities scale scores, but this was attenuated to a nonsignificant level in the final adjusted models.

**Table 2.** Longitudinal Associations Between Parent-Reported Sedentary Behavior Among Children at Age 7 Years and Score on the British Ability Scale Verbal Similarities Scale at Age 11 Years, Millennium Cohort Study, 2001–2013

Sedentary Behavior and Level	No.	Model 1^a^		Model 2^b^		Model 3^c^	
		B	95% CI	B	95% CI	B	95% CI
Television viewing							
Low	1,739	0	Referent	0	Referent	0	Referent
Medium	5,510	−0.5	−1.0, 0.1	0.0	−0.5, 0.5	0.1	−0.3, 0.6
High	1,213	−1.7	−2.4, −1.0	−0.6	−1.2, 0.1	−0.1	−0.8, 0.5
Playing computer/electronic games							
Low	885	0	Referent	0	Referent	0	Referent
Medium	4,608	1.3	0.6, 2.0	0.3	−0.3, 0.9	0.1	−0.5, 0.7
High	2,969	0.2	−0.6, 0.9	−0.1	−0.8, 0.5	−0.1	−0.7, 0.6
Reading							
Low	3,126	0	Referent	0	Referent	0	Referent
Medium	1,930	1.0	0.4, 1.5	0.0	−0.5, 0.5	−0.2	−0.7, 0.3
High	3,406	2.6	2.1, 3.1	0.6	0.1, 1.0	0.4	−0.1, 0.8
Doing arts and crafts							
Low	2,035	0	Referent	0	Referent	0	Referent
Medium	2,841	1.0	0.5, 1.6	0.1	−0.4, 0.6	0.2	−0.3, 0.7
High	3,586	0.7	0.2, 1.2	0.2	−0.2, 0.7	0.4	−0.1, 0.9
Doing homework							
Low	2,925	0	Referent	0	Referent	0	Referent
Medium	2,757	0.7	0.2, 1.2	0.3	−0.2, 0.7	0.1	−0.4, 0.5
High	2,780	0.9	0.4, 1.4	0.7	0.2, 1.1	0.5	0.0, 0.9

Abbreviation: CI, confidence interval.

^a^ Adjusted for sex.

^b^ Adjusted for the variables in model 1 and the composite score of previous British Ability Scale assessments at ages 3–7 years.

^c^ Adjusted for the variables in model 2 and ethnicity, maternal age, maternal qualifications, income, smoking status during pregnancy, and Strengths and Difficulties Questionnaire score.

**Table 3.** Longitudinal Associations Between Parent-Reported Physical Activity Among Children at Age 7 Years and Score on the British Ability Scale Verbal Similarities Scale at Age 11 Years, Millennium Cohort Study, 2001–2013

Activity and Level	No.	Model 1^a^		Model 2^b^		Model 3^c^	
		B	95% CI	B	95% CI	B	95% CI
Attendance at sports/PA clubs							
Low	2,333	0	Referent	0	Referent	0	Referent
Medium	2,299	2.0	1.5, 2.6	0.6	0.1, 1.1	0.3	−0.2, 0.8
High	3,830	3.4	2.9, 3.9	1.2	0.7, 1.6	0.6	0.2, 1.1
Sports/PA with siblings							
Low	945	0	Referent	0	Referent	0	Referent
Medium	1,887	0.8	0.1, 1.6	−0.1	0.6, 0.8	−0.1	−0.8, 0.6
High	5,630	−0.4	−1.0, 0.3	−0.7	−1.3, −0.1	−0.6	−1.2, 0.0
Sports/PA with parents							
Low	1,824						
Medium	3,090	1.1	0.6, 1.7	0.5	0.0, 1.0	0.4	−0.2, 0.9
High	3,548	0.4	−0.1, 1.0	0.1	−0.4, 0.6	0.2	−0.3, 0.6
Park/playground visits							
Low	1,513	0	Referent	0	Referent	0	Referent
Medium	2,690	0.7	0.1, 1.3	0.2	−0.4, 0.7	0.2	−0.4, 0.7
High	4,259	−0.5	0.1, 1.0	0.3	−0.2, 0.8	0.3	−0.2, 0.8

Abbreviations: CI, confidence interval; PA, physical activity.

^a^ Adjusted for sex.

^b^ Adjusted for the variables in model 1 and the composite score of previous BAS assessments at ages 3–7 years.

^c^ Adjusted for the variables in model 2 and ethnicity, maternal age, maternal qualifications, income, smoking status during pregnancy, and Strengths and Difficulties Questionnaire score.

Objectively measured sedentary time at age 7 years was positively associated with BAS Verbal Similarities scale score at age 11 years after final adjustment (see Table 4). Light activity was inversely associated with cognitive scores, but there were no observed associations with MVPA. The association between objective sedentary time and cognitive scores was not modified by self-reported reading time (Web Table 3). The association with time in objectively assessed MVPA remained nonsignificant after stratifying by attendance at sports/physical activity clubs (Web Table 4). Further stratification by sex revealed that the positive association between sedentary time and cognitive scores was largely driven by girls (B = 1.1; 95% confidence interval: 0.2, 2.0) rather than boys (B = 0.3; 95% confidence interval: −0.7, 1.3) (Web Table 5). Time in MVPA was inversely associated with cognitive scores in girls (B = −1.1; 95% confidence interval: −2.0, −0.3) but not in boys (B = 0.4; 95% confidence interval: −0.6, 1.2).

**Table 4.** Longitudinal Associations of Accelerometry-Derived Sedentary Time, Light-Intensity Activity, and Moderate-to-Vigorous Physical Activity at Age 7 Years With Score on the British Ability Scale Verbal Similarities Scale at Age 11 Years, Millennium Cohort Study, 2001–2013

Variable	No.	Model 1^a^		Model 2^b^		Model 3^c^	
		B	95% CI	B	95% CI	B	95% CI
Sedentary time							
Low	1,596	0	Referent	0	Referent	0	Referent
Medium	1,565	1.2	0.6, 1.9	0.7	0.1, 1.3	0.5	0.0, 1.1
High	1,563	1.7	1.0, 2.4	1.0	0.3, 1.7	0.8	0.1, 1.4
Light PA time							
Low	1,593	0	Referent	0	Referent	0	Referent
Medium	1,607	−0.6	−1.2, 0.1	−0.5	−1.0, 0.1	−0.4	−0.9, 0.2
High	1,524	−1.3	−2.0, −0.6	−0.9	−1.5, −0.3	−0.7	−1.3, −0.1
MVPA time							
Low	1,575	0	Referent	0	Referent	0	Referent
Medium	1,598	−0.2	−0.9, 0.4	−0.2	−0.7, 0.4	−0.1	−0.7, 0.5
High	1,551	−1.0	−1.7, −0.3	−0.6	−1.2, 0.0	−0.4	−1.0, 0.2

Abbreviations: CI, confidence interval; MVPA, moderate-to-vigorous physical activity; PA, physical activity.

^a^ Adjusted for sex and actigraph weartime.

^b^ Adjusted for the variables in model 1 and the composite score of previous British Ability Scale assessments at ages 3–7 years.

^c^ Adjusted for the variables in model 2 and ethnicity, maternal age, maternal qualifications, income, smoking status during pregnancy and Strengths and Difficulties Questionnaire score.

## DISCUSSION

We found that specific domains of both physical activity and sedentary behavior were positively associated with cognitive function. Objectively measured sedentary time was also positively associated with cognitive function in this sample of children in the United Kingdom.

### Physical activity and cognitive function

Attendance at sports/physical activity clubs at 7 years of age was positively associated with cognitive function at 11 years of age. Conversely, engaging in sports/physical activity with siblings was inversely associated. There have been several studies to date in which researchers have examined the association of physical activity with cognitive function, but the associations between specific domains of activity and cognition are not understood. In a meta-analysis, Sibley and Etnier (1) reported no significant differences in the associations of different types of activity (i.e., resistance training, physical education classes, aerobic exercise, and perceptual-motor training) with measures of cognition. In contrast, investigators in another study found that coordinative exercise elicited greater improvements in attention and concentration than did a “normal” sports lesson (14). It is believed that coordinative exercise engages more frontal-dependent cognitive processes than do basic repetitive exercises. Tomporowski et al. (35) support this idea by finding similar cognitive performance in overweight children after treadmill walking compared with after watching a video. In a recent review, Best (15) suggested that the association between physical activity and executive function is dependent on the content of the activity, with activities that involve interaction with others, complex movements, and strategic and goal-directed behavior providing the most benefit. Activities at sports clubs are structured and are likely to include the types of activities that are reportedly beneficial to cognitive function, whereas activities conducted in children's discretionary time, such as sports/physical activity with siblings or parents, are less likely to include them, which may explain the present findings.

Objectively measured time spent engaging in MVPA at age 7 years was not associated with cognitive function at age 11 years, which suggests that participation in MVPA at a young age does not impair cognitive development in the long term. However, when the models were run separately for girls and boys, MVPA in girls was negatively associated with cognitive function. Differences between sexes may be explained by the context of time spent engaging in MVPA. It is possible that girls' MVPA may consist of less cognitively engaging components than does boys' MVPA. Differences by sex have also been shown in previous research. For example, Booth et al. (21) found that the positive relationship between MVPA and attention was stronger in males. These results may simply reflect the types of physical activities and sedentary behaviors in which girls and boys tend to engage. In a small number of studies to date, researchers have found a positive association between objectively measured physical activity and cognitive function in youths (12, 21), but this is not supported by the present study. A plausible explanation for this finding may be that the predominant physical activities performed in the present sample, as measured by accelerometry, do not consist of cognitively engaging components (e.g., activities that are challenging and goal-orientated and that involve complex movements). Physical activity as measured by accelerometry includes all types of activities, including those with less cognitively stimulating content. It may be that participating in structured physical activities is more beneficial for cognition than participating in less structured activity, although our data were not able to fully confirm this.

Alternatively, the discrepancies between our results and those from other recent studies may be explained by the different measures used. The BAS assessments used in the present study were primarily used to assess verbal abilities, whereas those from other studies were used to assess other domains of cognition. For example, in previous research, investigators found favorable associations of MVPA with attention (21) and reaction time (12) in children of similar ages. Furthermore, because of the nature of the accelerometry data collection, we are unable to quantify the association of acute bouts of physical activity with cognitive function. The timing of physical activity bouts may be a critical factor determining these associations (36).

### Sedentary behavior and cognitive function

Participation in homework at age 7 years was positively associated with cognitive function at age 11 years. These associations may be explained by the high levels of cognitive engagement required for this type of behavior. Reading was also positively associated with cognitive function in the initial and intermediate models. The association decreased substantially after adjustment for previous cognitive function at ages 3, 5, and 7 years and was reduced to a nonsignificant level after full adjustment. The results suggest that if there is a beneficial association of reading with cognitive function, it occurs primarily before 7 years of age. In the initial models, television viewing was negatively associated with cognitive function at age 11 years, which is in line with the majority of previous research in children and adolescents (16, 37). However, after controlling for previous cognitive function, we found that television viewing at 7 years of age was no longer associated with cognitive function at 11 years of age. The key strength of the present study was our ability to robustly control for cognitive development several years before baseline, which has been a major limitation of previous studies. Our data suggest that if there is a negative association of television with cognition, it occurs during early childhood, and therefore adjustment for prior and baseline cognition might in effect have adjusted out the association with television viewing in the present analyses. Furthermore, early childhood cognitive function may play a role in driving children's television viewing because children with lower cognitive ability may find other activities more challenging. Previous evidence suggests that television viewing displaces time spent engaging in activities that are beneficial for cognitive function, such as arts and crafts and homework (38). There has been some research to date indicating that there are negative associations between other forms of screen time, such as computer/video game usage, and cognitive function, but this is not supported in the present study (12). Another body of evidence suggests that computer/video game usage may actually be beneficial to certain domains of cognition (20, 39).

Higher levels of objectively measured sedentary time at age 7 years were associated with better cognitive performance at age 11 years. Sedentary time at age 7 years was the strongest predictor of cognitive function at age 11 years after full adjustment. The results from our contextual analyses would suggest that this association is driven by sedentary behaviors that require high levels of cognitive engagement, such as doing homework. These data are in agreement with those from a recent study in which investigators found positive associations between objectively measured sedentary time and attention (12). After stratifying by sex, we demonstrated that sedentary time was more strongly associated with cognitive function in girls. Compared with sedentary time in boys, girls' sedentary time may be spent doing more beneficial activities (i.e., arts and crafts and homework). Recent research has shown that children with higher socioeconomic status record greater overall sedentary time yet spend less time watching television than do children with lower socioeconomic status (40). Because the children in our accelerometry sample typically came from wealthier families and had parents with higher levels of education than did those excluded from this part of the analysis (see Web Table 2), this may have introduced bias to the results, with potentially a higher proportion of our sample spending more time in non-television sedentary behaviors than a truly representative sample.

The major strengths of the present study are the longitudinal design, which allowed us to robustly control for cognitive development several years before baseline; the large and representative sample; and the use of objective and self-reported measures of physical activity and sedentary behavior. One limitation is that important facets of cognitive function, such as attention and memory, which may be affected differently by physical activity and sedentary behavior, were not explored in this study. Future research using a similar design should seek to understand how physical activity and sedentary behavior are associated with these aspects of cognitive function. Another limitation is that accelerometers only provide data on a single week, which may not be a true reflection of habitual behavior. They are also unable to capture data for certain activities, such as cycling and swimming; therefore, objectively measured activity levels may be underestimated. In addition, recent data have shown that, when measured objectively, some children may be predominantly sedentary during some organized sports yet demonstrate high accelerometry counts during screen time (41).

In conclusion, the associations of physical activity and sedentary behavior with cognitive function appear to be context-specific in young people. Interventions aiming to increase cognitive performance should focus on increasing participation in specific contexts of sedentary behavior and physical activity, such as structured sports and homework, rather than aiming to change overall activity or sedentary levels.

## Supplementary Material

Web Material
