# Supplementary material for: Context-Specific Associations of Physical Activity and Sedentary Behavior With Cognition in Children
Source: Am J Epidemiol. 2016 May 24;183(12):1075–82. doi: 10.1093/aje/kww031 (PMC4908213; doi:10.1093/aje/kww031)
Supplement: Web Material [file supp_183_12_1075__index.html]

Context-Specific Associations of Physical Activity and Sedentary Behavior With Cognition in Children — Web Material 

# Context-Specific Associations of Physical Activity and Sedentary Behavior With Cognition in Children

## Web Material

Web Material

- Web Material - Pdf file
