# Supplementary material for: Context-Specific Associations of Physical Activity and Sedentary Behavior With Cognition in Children
Source: Am J Epidemiol. 2016 May 24;183(12):1075–82. doi: 10.1093/aje/kww031 (PMC4908213; doi:10.1093/aje/kww031)
Supplement: Web Material [file supp_kww031_kww031supp.pdf]

Web Figure 1

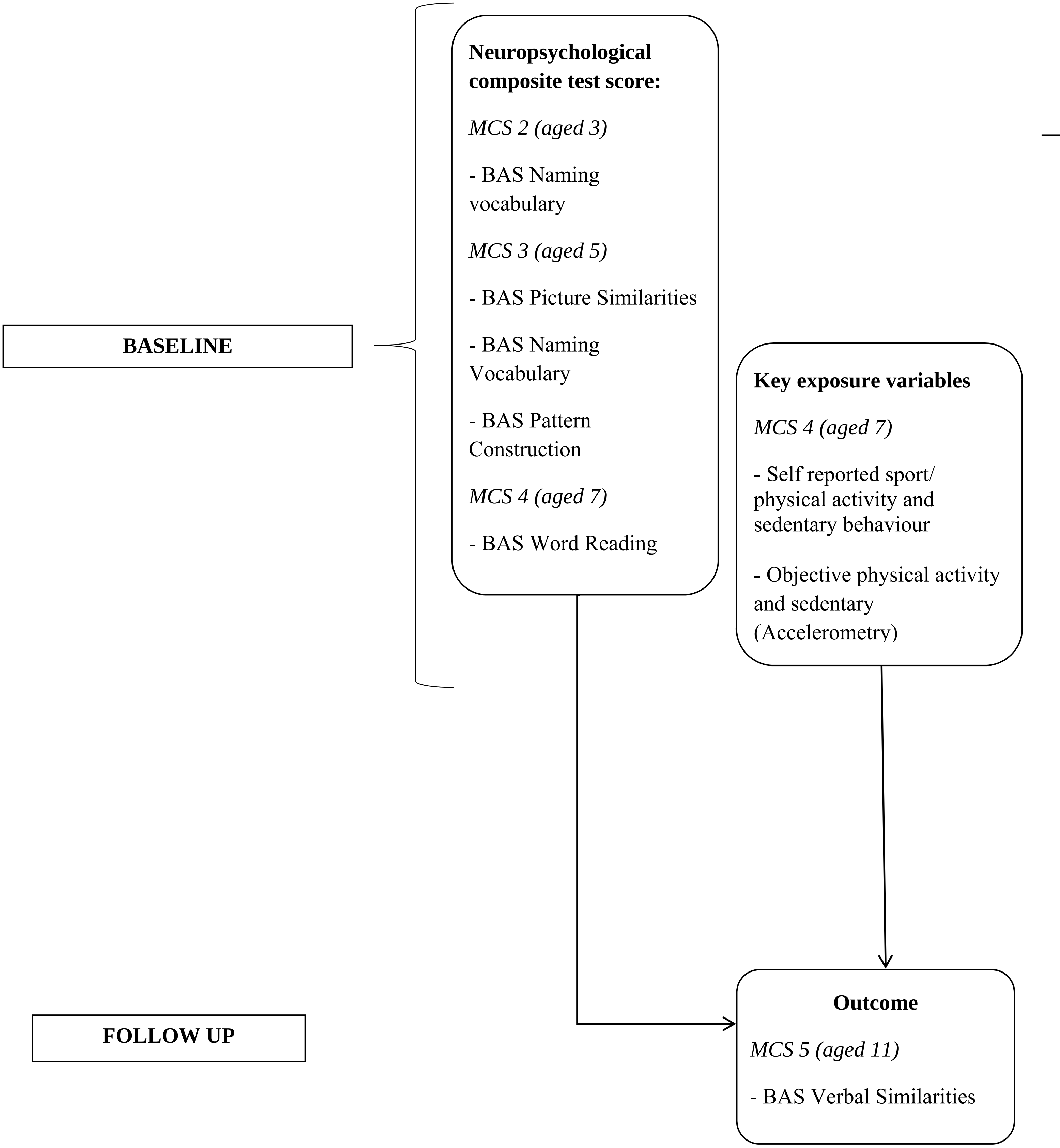
BASELINE
Neuropsychological composite test score:
MCS 2 (aged 3)
- BAS Naming vocabulary
MCS 3 (aged 5)
- BAS Picture Similarities
- BAS Naming Vocabulary
- BAS Pattern Construction
MCS 4 (aged 7)
- BAS Word Reading
Key exposure variables
MCS 4 (aged 7)
- Self reported sport/ physical activity and sedentary behaviour
- Objective physical activity and sedentary (Accelerometry)
FOLLOW UP
Outcome
MCS 5 (aged 11)
- BAS Verbal Similarities

Web Table 1. Correlation between BAS cognitive scores at ages 3 to 7 in the Millennium Cohort Study, 2001-2013

	**Naming vocabulary (age 3)**	**Naming vocabulary (age 5)**	**Picture Similarity (age 5)**	**Pattern construction (age 5)**	**Pattern construction (age 7)**	**Word reading (age 7)**
Naming vocabulary (age 3)	1	–	–	–	–	–
Naming vocabulary (age 5)	0.6	1	–	–	–	–
Picture Similarity (age 5)	0.2	0.3	1	–	–	–
Pattern construction (age 5)	0.3	0.4	0.4	1	–	–
Pattern construction (age 7)	0.3	0.3	0.3	0.6	1	–
Word reading (age 7)	0.3	0.4	0.2	0.3	0.3	1

Web Table 2. Differences in characteristics between accelerometry sample and remaining cohort at age 7 in the Millennium Cohort Study, 2001-2013

	**Acclerometry sample (n=4724)**		**Remaining sample (n=3738)**	
	**%**	**Mean (SD)**	**%**	**Mean (SD)**
Male sex	48.8		51.5*	
White British	90.6		85.3**	
Maternal age, years		37.2 (5.5)		35.8 (5.9)**
Maternal qualifications, ≥ National Vocational Qualification level 4	47.3		35.1**	
Income, in poverty	18.9		30.0**	
Smoked during pregnancy	26.8		37.0**	
Strengths and Difficulties Questionnaire score		6.8 (4.7)		8.0 (5.3)**
BAS verbal similarities score		60.5 (9.0)		58.3 (10.0)**

Abbreviations: SD, standard deviation, BAS, British Ability Scale. *p<0.05; **p<0.001

Web Table 3. Associations between accelerometry-derived sedentary time, light intensity activity and MVPA at age 7 and BAS verbal similarities score at age 11 stratified by time spent reading at age 7 (low vs. high) in the Millennium Cohort Study, 2001-2013

		**Low (n=2741)**			**High (n=1983)**	
	**No.**	**B[a]**	**95% CI**	**N**	**B[a]**	**95% CI**
Sedentary time						
Low	978	REF		618	REF	
Med	895	0.9	0.1, 1.7	670	0.1	-0.9, 0.9
High	868	0.7	-0.2, 1.7	695	0.8	-0.2, 1.8
Light PA time						
Low	872	REF		721	REF	
Med	917	-0.5	-1.3, 0.3	690	-0.3	-1.1, 0.6
High	952	-0.7	-1.5, 0.1	572	-0.6	-1.6, 0.3
MVPA time						
Low	809	REF		766	REF	
Med	906	-0.1	-0.9, 0.7	692	-0.1	-0.9, 0.8
High	1026	-0.1	-0.9, 0.8	525	-0.9	-1.8, 0.1

Abbreviations: MVPA, moderate-to-vigorous physical activity, BAS, British Ability Scale, CI, Confidence Interval, REF, Reference Category, PA, physical activity.
[a]Adjusted for actigraph weartime, the composite score of previous BAS assessments at ages 3 to 7, ethnicity, maternal age, maternal qualifications, income, smoking status during pregnancy and Strengths and Difficulties Questionnaire score.

Web Table 4. Associations between accelerometry-derived sedentary time, light intensity activity and MVPA at age 7 and BAS verbal similarities score at age 11 stratified by attendance at sports/PA clubs at age 7 (low vs. high) in the Millennium Cohort Study, 2001-2013

		**Low (n=2307)**			**High (n=2417)**	
	**No.**	**B[a]**	**95% CI**	**N**	**B[a]**	**95% CI**
Sedentary time						
Low	753	REF		843	REF	
Med	748	1.1	0.2, 1.9	817	0.1	-0.7, 0.9
High	806	0.7	-0.3, 1.7	757	1.0	0.1, 1.8
Light PA time						
Low	782	REF		811	REF	
Med	780	-0.1	-0.9, 0.8	827	-0.6	-1.4, 0.2
High	745	-0.7	-1.6, 0.3	779	-0.8	-1.7, 0.0
MVPA time						
Low	800	REF		775	REF	
Med	795	-0.4	-1.2, 0.5	803	0.2	-0.6, 1.0
High	712	-0.7	-1.7, 0.2	839	-0.2	-1.0, 0.7

Abbreviations: MVPA, moderate-to-vigorous physical activity, BAS, British Ability Scale, CI, Confidence Interval, REF, Reference Category, PA, physical activity.
[a]Adjusted for actigraph weartime, the composite score of previous BAS assessments at ages 3 to 7, ethnicity, maternal age, maternal qualifications, income, smoking status during pregnancy and Strengths and Difficulties Questionnaire score.

Web Table 5. Associations between accelerometry-derived sedentary time, light intensity activity and MVPA at age 7 and BAS verbal similarities score at age 11 stratified by sex in the Millennium Cohort Study, 2001-2013

		**Boys (n=2303)**		**Girls (n=2421)**		
	**No.**	**B[a]**	**95% CI**	**N**	**B[a]**	**95% CI**
Sedentary time						
Low	867	REF		729	REF	
Med	735	0.4	-0.4, 1.2	830	0.7	-0.2, 1.5
High	701	0.3	-0.7, 1.3	862	1.1	0.2, 2.0
Light PA time						
Low	734	REF		859	REF	
Med	788	-0.4	-1.3, 0.4	819	-0.2	-1.0, 0.6
High	781	-0.1	-1.0, 0.8	743	-1.2	-2.1, -0.4
MVPA time						
Low	489	REF		1086	REF	
Med	750	0.6	-0.4, 1.5	848	-0.4	-1.2, 0.3
High	1064	0.4	-0.6, 1.2	487	-1.1	-2.0, -0.3

Abbreviations: MVPA, moderate-to-vigorous physical activity, BAS, British Ability Scale, CI, Confidence Interval, REF, Reference Category, PA, physical activity.
[a]Adjusted for actigraph weartime, the composite score of previous BAS assessments at ages 3 to 7, ethnicity, maternal age, maternal qualifications, income, smoking status during pregnancy and Strengths and Difficulties Questionnaire score.
